# Validation of two multiplex platforms to quantify circulating markers of inflammation and endothelial injury in severe infection

**DOI:** 10.1371/journal.pone.0175130

**Published:** 2017-04-18

**Authors:** Aleksandra Leligdowicz, Andrea L. Conroy, Michael Hawkes, Kathleen Zhong, Gerald Lebovic, Michael A. Matthay, Kevin C. Kain

**Affiliations:** 1 Department of Medicine, University of Toronto, Toronto, Canada; 2 Sandra A. Rotman Laboratories, Sandra Rotman Centre for Global Health, University Health Network, Toronto, Canada; 3 Department of Pediatrics, Indiana University School of Medicine, Indianapolis, United States of America; 4 Division of Pediatric Infectious Diseases, University of Alberta, Edmonton, Canada; 5 Applied Health Research Centre, The HUB, Li Ka Shing Knowledge Institute, University of Toronto, Toronto, Canada; 6 Departments of Medicine and Anesthesia, University of California, San Francisco, United States of America; 7 Cardiovascular Research Institute, University of California, San Francisco, United States of America; California State University Fresno, UNITED STATES

## Abstract

Biomarkers can prognosticate outcome and enable risk-stratification. In severe infection, focusing on multiple markers reflecting pathophysiological mechanisms of organ injury could enhance management and pathway-directed therapeutics. Limited data exist on the performance of multiplex biomarker platforms. Our goal was to compare endothelial and immune activation biomarkers in severe pediatric infections using two multiplex platforms. Frozen plasma from 410 children presenting to the Jinja Regional Hospital in Uganda with suspected infection was used to measure biomarkers of endothelial (Angiopoietin-2, sFlt-1, sVCAM-1, sICAM-1) and immune (IL-6, IP-10, sTNFR-1, CHI3L1) activation. Two multiplex platforms (Luminex®, Ella^TM^) based on monoclonal antibody sandwich immunoassays using biotin-streptavidin conjugate chemistry were selected with reagents from R&D Systems. The two platforms differed in ease and time of completion, number of samples per assay, and dynamic concentration range. Intra-assay variability assessed using a coefficient of variation (CV%) was 2.2–3.4 for Luminex® and 1.2–2.9 for Ella^TM^. Correlations for biomarker concentrations within dynamic range of both platforms were best for IL-6 (ρ = 0.96, p<0.0001), IP-10 (ρ = 0.94, p<0.0001) and sFlt-1 (ρ = 0.94, p<0.0001). Agreement between concentrations obtained by both methods assessed by the Bland-Altman test varied, with best agreement for CHI3L1. Our data suggest that biomarkers of endothelial and immune activation can be readily measured with multiplex platforms. Luminex® and Ella^TM^ produced reliable results with excellent CV% values. The Ella^TM^ platform was more automated and completed in 75 minutes, potentially compatible with near-patient use. Trends in concentrations obtained by these methods were highly correlated, although absolute values varied, suggesting caution is required when comparing data from different multiplex platforms.

## Introduction

The search for novel biological markers to predict response to therapies, prognosticate outcome, or assist in patient enrollment in clinical therapeutic trials is quickly evolving [1]. In the context of life-threatening infection, many biomarkers have been proposed to improve the discriminatory ability to achieve these goals [2, 3]. Emphasizing markers of pathophysiological pathways involved in severe infections and focusing on multiplex platforms with near-patient or point-of-care potential, could accelerate the development of precision medicine tools for life-threatening infections [4, 5].

The third iteration of the international consensus definitions for sepsis and septic shock [6] acknowledged that while multiple candidate biomarkers have been evaluated, robust validation is required prior to incorporating them into a clinical definition of sepsis. However, many challenges exist with the validation of biomarkers, including inconsistency in the biological reagents used, the detection platforms utilized, the combinations and permutations of markers tested, the diversity of patient cohorts from which samples are derived, and the statistical tests used to analyze the results.

Of the many pathophysiologic pathways that may contribute to the high morbidity and mortality of severe infections, endothelial [7, 8] and immune activation [9, 10] have been studied extensively. Key biomarkers of endothelial activation include the Angiopoietin-Tie2 axis [11], the soluble variant of the vascular endothelial growth factor, a receptor known as soluble fms-like tyrosine kinase-1 (sFlt-1) [12, 13], soluble vascular cell adhesion molecule-1 (sVCAM-1) and soluble intercellular adhesion molecule-1 (sICAM-1) [14, 15]. Multiple markers of inflammation have been identified and among these interleukin 6 (IL-6) [16], interferon-gamma-inducible protein-10 (IP-10, CXCL10) [17], chitinase-3-like-1 protein (CHI3L1) [18] and soluble tumor necrosis factor receptor-1 (sTNFR-1) [15, 19] have been correlated with severity of illness and clinical outcome in sepsis as well as other critical care illnesses such as the acute respiratory distress syndrome, usually caused by severe infection [20, 21].

The quantification of plasma proteins still largely relies on the use of enzyme-linked immunosorbent (ELISA)-based assays [22]. In the past decade, the simultaneous detection of multiple distinct proteins was enabled by using highly specific capture and detection monoclonal antibodies [23–26]. The detection antibodies can be conjugated to different indicators, allowing for the quantification of properties such as optical density, electrochemiluminescence, chemiluminescence, or fluorescence intensity. The property of the indicator as well as the instrument used to detect it lead to variation in the sensitivity and dynamic range of the assay, the number of analytes that can be simultaneously analyzed, and reagent cost. Based on its established performance over the past 20 years, we selected the magnetic microsphere-based Luminex® platform [26–29] and compared it to a novel, fully automated microfluidics-based platform, Ella^TM^ [30–32].

The main goal of this study was to use reagents prepared by a single manufacturer (R&D Systems) to compare concentrations of previously identified biomarkers of endothelial and immune activation in patients with severe infection measured by two different multiplex platforms. Secondly, we address the challenge of using appropriate statistical tests to compare the performance characteristics of two platforms, with an analytic focus on the ease of use, assay dynamic range, intra-assay variability, and agreement between biomarker concentrations computed by each platform. Our findings have important implications for future near-patient and point-of-care biomarker quantification in life-threatening infection.

## Materials and methods

### Patient sample selection

A previously described prospective cohort of 2,085 consecutive febrile children aged 2 months to 5 years old who presented to the Jinja Regional Referral Hospital in Uganda, 4.7% of whom died during hospital admission, [33] was used to generate a subcohort of patients for this study. Subjects for this nested case cohort design were selected by randomly sampling 18% of the whole cohort and adding all non-sampled deaths that occurred during hospital admission. The subcohort included 410 children, 99 of whom died during hospital admission and 301 who survived to hospital discharge.

Ethical approval was obtained from Uganda National Council for Science and Technology, Makerere University Research Ethics Committee in Uganda, and the University Health Network. Written informed consent for all study participants was provided by the parent or caregiver.

### Plasma sample preparation

Up to 1 ml of whole blood was collected by venipuncture and anticoagulated using ethylenediaminetetraacetic acid (EDTA). Blood was centrifuged within 4 hours of sample collection and plasma was frozen at -80°C without freeze-thaw until analyzed. Samples were thawed overnight at 4°C and aliquoted at room temperature immediately prior to assay performance.

### Luminex® platform protocol

Reagents for Luminex® assays were custom developed by R&D Systems. Thirteen biomarkers were selected and divided into 2 panels based on relative plasma abundance and assay dynamic range. Panel 1 included the following 5 high-abundance biomarkers tested at a dilution of 1:30: sVCAM-1, sICAM-1, sTNFR-1, CHI3L1, Cystatin C. Panel 2 included the following 8 low-abundance biomarkers tested at a dilution of 1:3: Angiopoeitin-2 (Ang-2), sFlt-1, IL-6, IP-10, Ang-1, IL-8, sTREM-1, Granzyme B. The 5 biomarkers that did not overlap between the platforms are presented in **S1 Table**. Unfiltered plasma was diluted using diluents supplied by the manufacturer. Each 96-well plate included 7-fold serial dilutions of standards tested in duplicate and 72 patient samples, 8 of which were tested in duplicate. A total of 6 batches of Luminex Panel 1 and 2 were necessary to complete analysis of 410 samples. Assays were performed according to manufacturer’s magnetic Luminex® screening assay protocol [34]. Briefly, a microparticle cocktail, diluted plasma, and biomarker standards were added to a 96-well plate. Following a 2-hour incubation, plates were washed and a biotin antibody cocktail was added. After a 1-hour incubation, plates were washed and streptavidin-Phycoerythrin (PE) was added for 30-min, followed by a final wash and resuspension in wash buffer. All incubations were done at room temperature on a microplate shaker at 800 rpm. Plates were read immediately on the MAGPIX® instrument and raw data were analyzed using the xPONENT® software. Values outside the lower limit of quantification were assigned a value of 1/3 of the lower limit of the standard curve.

### Ella^TM^ platform protocol

Reagents for the Simple Plex^TM^ Ella microfluidic platform (Protein Simple, CA, USA) were custom developed. Eight biomarkers that overlapped with the Luminex® platform were selected and divided into 2 panels based on relative plasma abundance and assay dynamic range. Panel 1 included the following 4 high-abundance biomarkers tested at a dilution of 1:100: sVCAM-1, sICAM-1, sTNFR1, CHI3L1. Panel 2 included the following 4 low-abundance biomarkers tested at a dilution of 1:10: Ang-2, sFlt-1, IL-6, IP-10. Plasma samples were analyzed on the same day and after a single thaw, the same as the corresponding samples analyzed using the Luminex® platform. Unfiltered plasma samples were diluted using diluents supplied by the manufacturer and assays were performed according to manufacturer’s protocol. Briefly, 50 μl of diluted plasma was added to the appropriate cartridge, followed by placement in to the Ella instrument, requiring no further user intervention. Each cartridge included a built-in lot-specific standard curve and samples were run as internal triplicates. This was accomplished by the presence of three nanorods present inside each individual channel corresponding to a single biomarker and each nanorod was coated with biomarker-specific capture monoclonal antibodies [30]. Detection monoclonal antibodies and streptavidin-DyLight650 conjugate as well as all washing steps were automatically performed by the instrument. Data were manually screened from each run for clogged nanorods or >20% discrepancy in readings between one of the three nanorods. Sixteen samples were accommodated by each cartridge, 5–6 cartridges were performed per day, and all assays were completed within 6 days. Raw data were analyzed using the SimplePlex Explorer software.

### Statistical analysis

Intra-assay variability was calculated using coefficient of variation (CV%) values [35] with the following formula: [standard deviation (σ)/mean (μ)]*100. The CV% values for the Luminex® platform were calculated from concertation values of duplicate samples from subjects who were part of the main cohort. 156 samples were included, spread across 20 plates. The CV% values for the Ella^TM^ platform were calculated from internal triplicate values of samples from all patients who were part of the subcohort. 410 samples were included, spread across 26 cartridges.

Platform comparisons were performed for values within dynamic range of both assays using three statistical tests [36, 37]. Spearman’s rank correlation was used to compute relationships of biomarker concentrations obtained by each platform. The paired Wilcoxon signed rank sum test was used to compare the median values obtained by each platform. The Bland-Altman method [38] was used to measure agreement between of Log_e_-transformed biomarker concentrations obtained by each platform. The variance of differences in concentration ([Log_e_ Luminex]-[Log_e_ Ella]) across the mean of concentration values (([Log_e_ Luminex]+[Log_e_ Ella])/2) was calculated using the Pitman's test [39]. Linear mixed effects models were used to calculate the 95% confidence intervals and p-values for the difference between Log_e_-transformed biomarker concentrations (bias), correcting for random Luminex batch effects for values within dynamic range of both assays.

Statistical analyses were performed using STATA v14.1 (StataCorp 2015). Graphical presentation was done using Excel (Microsoft 2016) and Prism v5.0a (GraphPad 2007).

## Results

### Biomarker assay performance

The Luminex® and Ella^TM^ platforms differed in ease of assay performance, number of samples analyzed per run, and time to perform each assay. Both platforms required optimization of plasma dilutions to ensure biomarker concentrations fell within the dynamic range of the assay.

The time to complete a Luminex assay was approximately 5 hours, accommodating up to 80 samples and up to 49 analytes per plate. The assay required manual preparation of serial dilutions of analyte standards, addition of pre-diluted samples, micro-particles, biotin-labeled antibodies, streptavidin substrate, three triplicate wash steps, and loading the plate into a MAGPIX instrument.

The time to complete an Ella assay was 1.25 hours, accommodating up to 16 samples and up to 4 analytes per plate. The Ella^TM^ platform requires loading the pre-diluted samples into a cartridge and inserting it into the instrument. No further user interface is required since the Ella instrument is fully automated, performs all assays in triplicate and uses internal microfluidics for completion of all immunoassay wash and detection steps.

### Biomarker assay limits of quantification and dynamic range

The Luminex® and Ella^TM^ platforms differed in lower limit of quantification (assay sensitivity) and biomarker dynamic range (**Table 1**). For samples measured using the Luminex® platform at an appropriate plasma dilution, 63.7–99.5% were within assay dynamic range. Majority of values outside of dynamic range were below the lower limit of quantification, suggesting lower assay sensitivity. For samples measured using the Ella platform at an appropriate plasma dilution, 94.6–100% were within assay dynamic range. The Ella^TM^ dynamic range was superior compared to Luminex® platform using reagents from R&D System especially, especially at the lower limits of quantification, and the only biomarker with values below the lower limit of assay quantification was IL-6. sICAM-1 values were not included in analyses as the sample dilution was inappropriate for reliable concentration quantification of values above the upper limit of the standard curve.

**Table 1:** Luminex® and Ella^TM^ multiplex platforms: dynamic range and limits of quantification for biomarkers of severe infection.

Biomarker	Luminex®				Ella^TM^			
	Dynamic Range (pg/mL)*	In dynamic range (%)	Below detection	Above detection	Dynamic Range (pg/mL)*	In dynamic range (%)	Below detection	Above detection
sVCAM-1	77,220–55,599,900	100% (410/410)	0	0	6,670–8,349,000	98.3% (403/410)	0	7
sICAM-1	73,590–54,410,070	92.4% (379/410)	31	0	390–1,563,000	48.9% (200/409)^#^	0	209
sTNFR-1	2,220–1,616,370	98.0% (402/410)	8	0	50–337,000	100% (410/410)	0	0
CHI3L1	33,210–2,690,910	63.7% (261/410)	138	11	290–3,850,500	99.0% (406/410)	0	4
Ang-2	402–98,319	99.5% (408/410)	0	2	72–378,100	100% (408/408)^#^	0	0
sFlt-1	216–52,725	79.5% (326/410)	84	0	26–46,500	100% (408/408)^#^	0	0
IL-6	15–10,269	70.2% (288/410)	102	20	5–42,770	94.6% (384/406)^#^	17	5
IP-10	12–2,769	89.3% (366/410)	0	44	4–9,200	96.8% (394/407)^#^	0	13

*Assay dynamic range adjusted for sample dilution (Luminex® platform: 1:30 dilution for sVCAM-1, sICAM-1, sTNFR-1, CHI3L1 and 1:3 dilution for Ang-2, sFlt-1, IL-6, IP-10; Ella^TM^ platform: 1:100 dilution for sVCAM-1, sICAM-1, sTNFR-1, CHI3L1 and 1:10 dilution for Ang-2, sFlt-1, IL-6, IP-10).

^#^Missing concentration values for Ella^TM^ platform due to blockage of either 2 or 3 nano-rods for the specified analytes.

### Intra-assay variability

**Fig 1:**
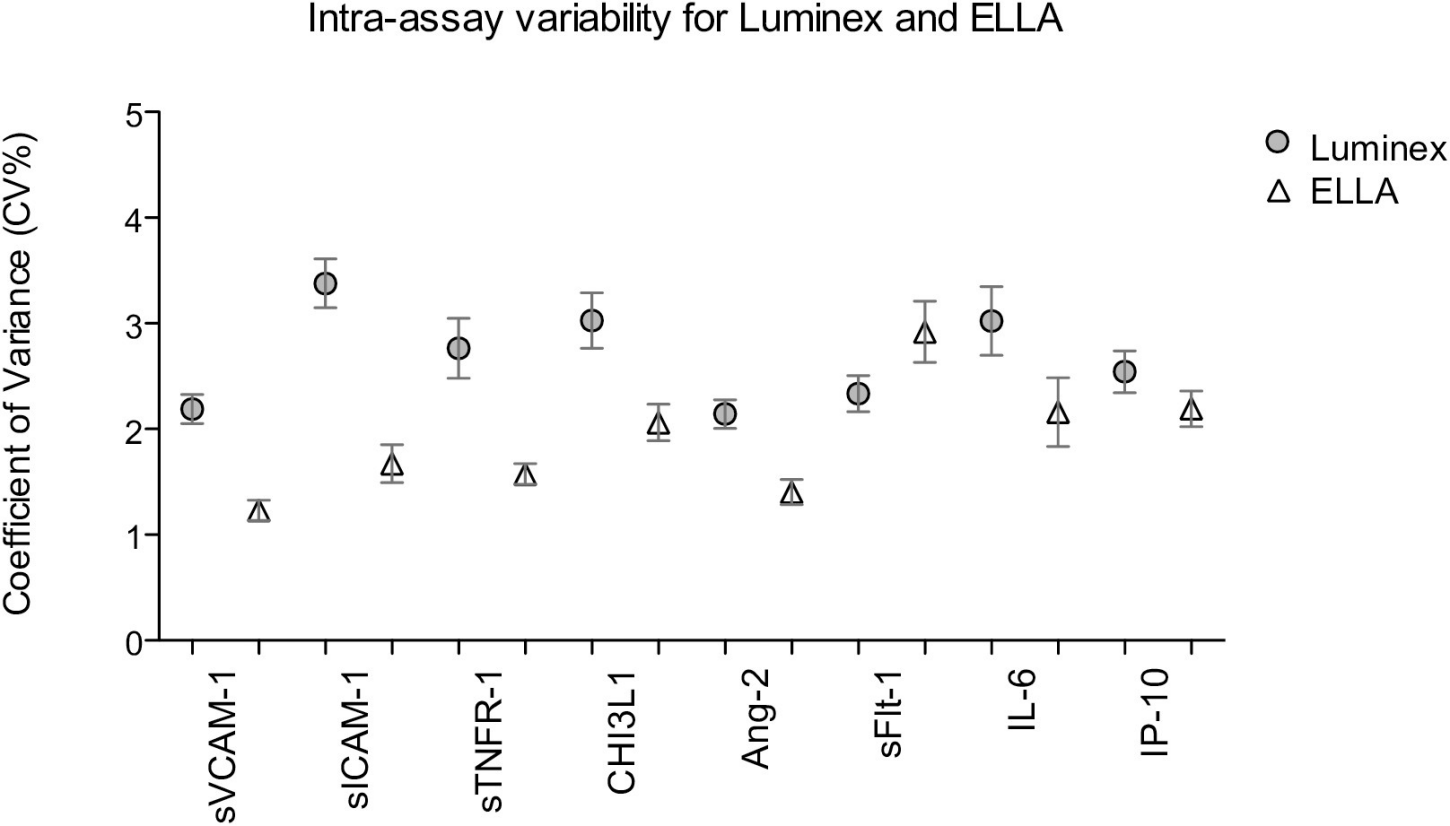
Intra-assay variability for biomarker concentrations determined using the Luminex® and Ella^TM^ platforms. Coefficient of variance (CV%) values for 156 sample assayed in duplicate by the Luminex® platform (shaded circles) and for 406–410 samples assayed by internal triplicate by the Ella^TM^ platform (open triangles). Analysis for sICAM-1 was excluded due to inappropriate dilution range. Graphs depict point estimates of the mean CV% and the corresponding 95% confidence intervals.

Intra-assay performance was calculated using coefficient of variation (CV%) values, to determine the precision of the concentration values obtained by both platforms [35]. The results are shown in **Fig 1**.

For the Luminex® platform, 156 random samples were assayed in duplicate. The CV% for the 8 biomarkers varied between 2.2–3.4. For the Ella^TM^ platform, all samples were run as internal triplicates and 2.7–12.3% of biomarkers were analyzed in duplicate due to an occlusion in one of three glass nanorods. For the 7 biomarkers analyzed at appropriate dilutions, the CV% varied between 1.2–2.9.

Inter-assay variability was determined by quantifying the same healthy control sample on each day of analysis (total of 6 days). The same sample was frozen in multiple aliquots and each aliquot was thawed once on the day of the assay. The quantified biomarkers for the healthy control sample were at the lower end or below the limit of quantification for both multiplex platforms. Therefore, inter-assay variability was greater than if a sample with biomarker concentration within the mid-zone of the standard curve was selected (**S2 Table**).

### Comparisons of concentration correlations

**Fig 2:**
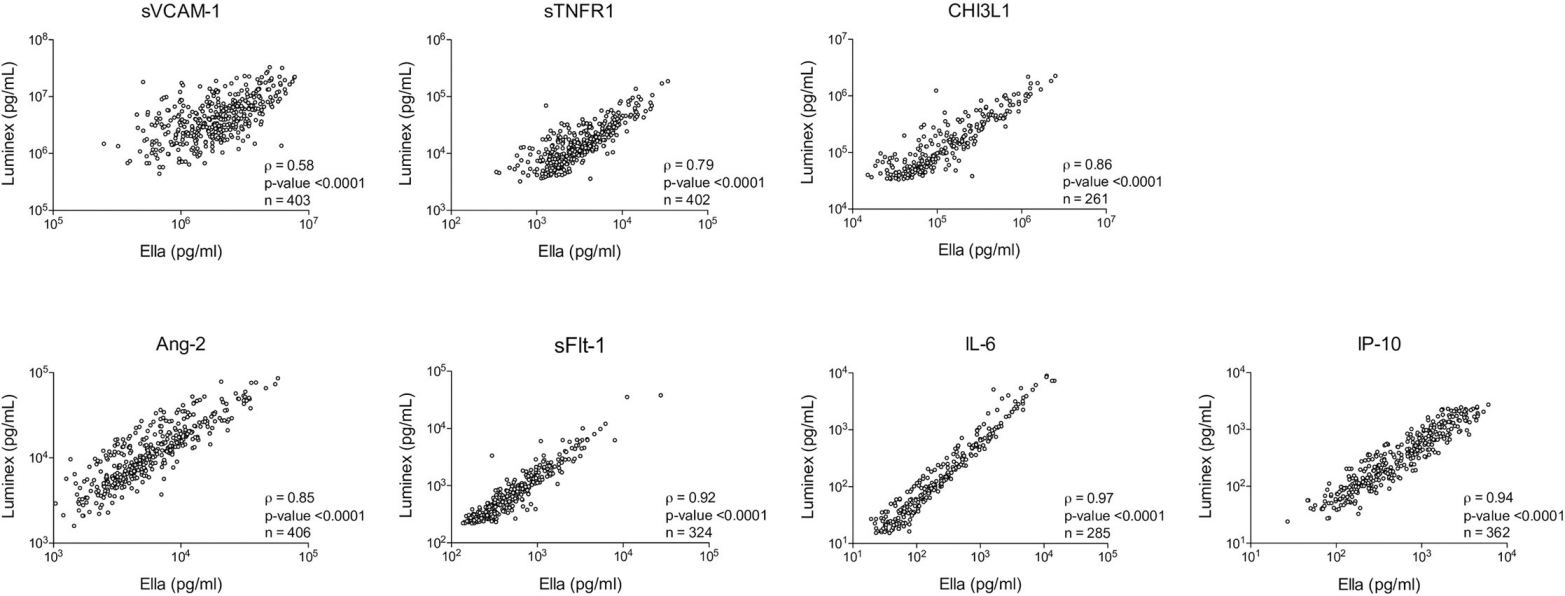
Relationship between raw untransformed concentration values obtained by the Luminex® compared to the Ella^TM^ platform. Spearman’s rho correlation coefficient (ρ) and p-values obtained using Spearman rank correlation for values (n) within assay dynamic range of both platforms.

Biomarker concentrations within dynamic range of both assays were compared using the Spearman correlation and paired Wilcoxon signed rank sum test. The strength of association for biomarker concentrations obtained using both platforms was excellent. For biomarkers quantified at appropriate dilutions, the strength of association varied between Spearman’s rho correlation coefficient (ρ) values of 0.79–0.97 (p-values <0.0001 for all analytes, **Fig 2**). The absolute median values for individual biomarkers quantified using the Luminex® and Ella^TM^ platforms varied (**Table 2**). For biomarker concentrations within the dynamic range of both multiplex platforms, there was no difference between the median CHI3L1 concentrations (p value = 0.99) but other biomarker concentrations varied significantly between the two multiplex platforms.

**Table 2:** Correlation between Luminex® and Ella^TM^ platform concentrations in pg/mL.

Biomarker	Median concentration (range) All samples		% in dynamic range of both platforms	Median concentration (range) In dynamic range of both platforms		p-value*
	Luminex®	Ella^TM^		Luminex®	Ella^TM^	
**sVCAM-1**	4,001,751 (439,807–49,800,000)	1,924,812 (249,888–14,900,000)	98.3% (403/410)	3,946,011 (439,807, 32,853,660)	1,910,286 (249,888, 7,778,393)	<0.0001
**sICAM-1**	786,044 (25,000–16,300,000)	1,584,729 (218,444–11,000,000)	45.5% (186/409)	654,608 (92,375, 6,021,572)	970,579 (218,444, 1,562,214)	<0.0001
**sTNFR-1**	12,592 (1,500–187,281)	2,942 (339–34,224)	98.0% (402/410)	12,771 (3,235, 187,281)	3,056 (339, 34,224)	<0.0001
**CHI3L1**	56,934 (11,000–3,496,523)	60,481 (2,198–5,628,866)	63.7% (261/410)	109,707 (33,225, 2,255,686)	105,574 (15,098, 2,499,149)	0.99
**Ang-2**	11,003 (1,597–151,000)	5,232 (859–132,764)	99.5% (406/408)	10,818 (1,597, 85,873)	5,184 (859, 57,703)	<0.0001
**sFlt-1**	483 (50–37,958)	391 (93.7–27,403)	79.4% (324/408)	682 (220, 37,958)	505 (136, 27,403)	<0.0001
**IL-6**	59 (0.1–20,601)	104 (0.8–194,571)	70.2% (285/406)	105 (15.3, 8,927)	168 (6.0, 14,592)	<0.0001
**IP-10**	393 (24.0–5,602)	713 (27.0–15,000)	88.9% (362/407)	322 (24, 2,737)	523 (27, 6,037)	<0.0001

*p-values computed using paired Wilcoxon signed rank sum test only for values within dynamic range of both multiplex platforms.

### Agreement between platforms

The Bland-Altman method [38] was used to assess agreement between Log_e_-transformed biomarker concentrations generated using the Luminex® and Ella^TM^ platforms. This graphical tool allows for depicting systematic bias between two methods of measurement and significant outlier values across the range of assay values.

**Fig 3:**
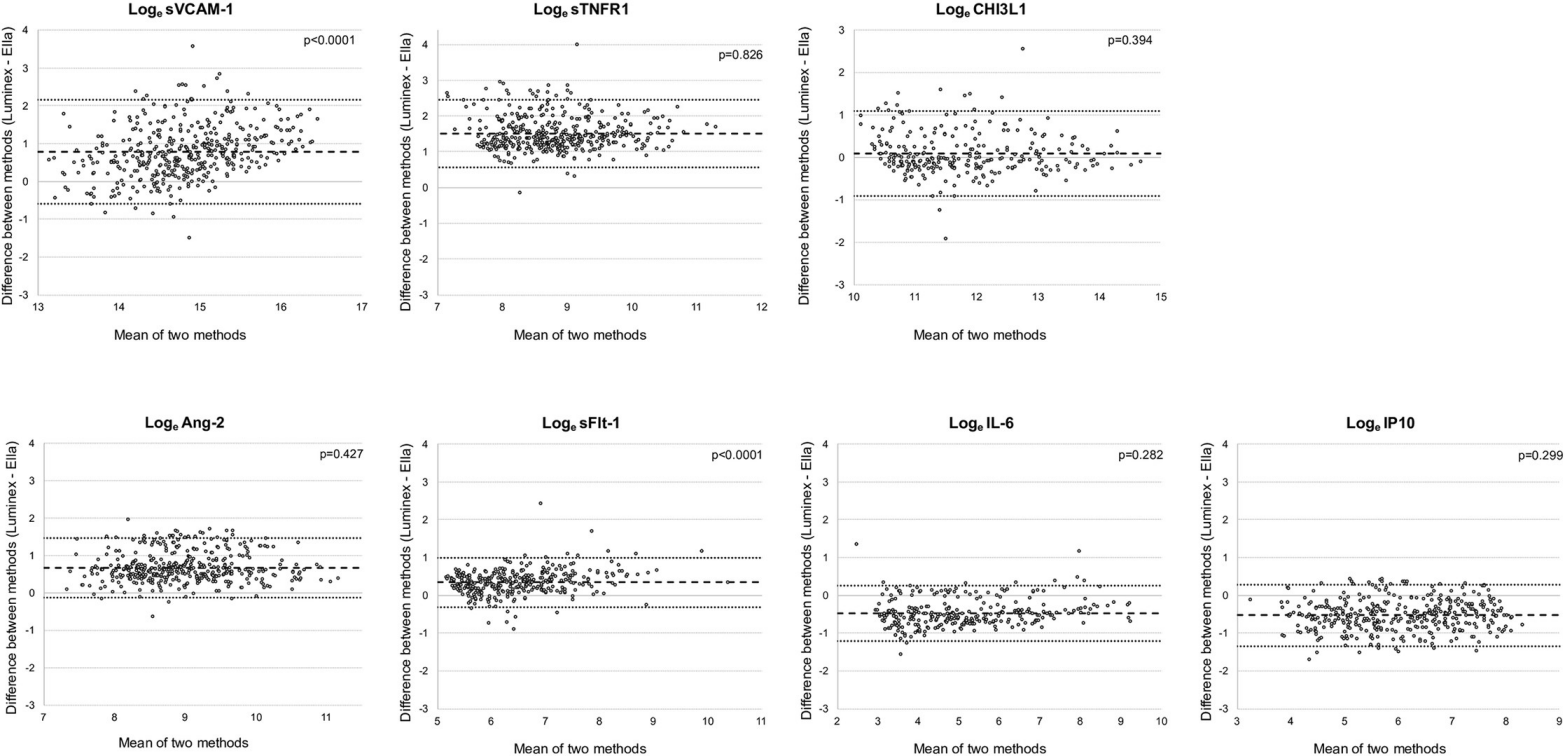
Bland-Altman plots comparing agreement between biomarker concentrations determined using the Luminex® and Ella^TM^ platforms. Upper and lower limits of agreement (dotted black lines) correspond to 2 standard deviations (SD) away from the mean difference (dashed black line). P-values represent Pitman's test of difference in variance, with a non-significant value indicating no variance across the range of mean values determined by both platforms. Analyses were performed on Log_e_-transformed biomarker concentrations that were within assay dynamic range of both platforms (summarized in Table 2).

As shown in **Fig 3**, the Log_e_-transformed concentrations of the 7 biomarkers showed varying patterns of agreement. These results were consistent with significant differences in median values demonstrated by paired Wilcoxon signed rank sum test shown in Table 2. A positive bias was indicative of higher concentration values measured by the Luminex® platform, while a negative bias higher concentrations obtained by the Ella^TM^ platform, A value close to zero meant that there was no difference between the two platforms for concentrations within the dynamic range of the two platforms. A significant bias towards one of the platforms was identified for all biomarkers, with the exception of CHI3L1 (**Table 3**). Using the Pitman's test of difference in variance, the bias for most biomarkers was consistent across the range of concentrations of both platforms, (p-values displayed in **Fig 3**).

**Table 3:** Bland-Altman bias representing difference in Log_e_-transformed biomarker concentrations computed by Luminex® compared to the Ella^TM^ platform for values within dynamic range of both assays.

Biomarker	Bias	p-value*
**sVCAM-1**	0.765 (0.549, 0.982)	<0.0001
**sTNFR-1**	1.492 (1.305, 1.680)	<0.0001
**CHI3L1**	0.047 (-0.133, 0.227)	0.61
**Ang-2**	0.688 (0.455, 0.920)	<0.0001
**sFlt-1**	0.338 (0.222, 0.455)	<0.0001
**IL-6**	-0.479 (-0.705, -0.253)	<0.0001
**IP-10**	-0.531 (-0.708, -0.354)	<0.0001

*p-values computed using linear mixed effects modeling with random batch effect to correct for possible Luminex batch influence (n = 6 batches).

## Discussion

In the past decade, the use of multiplex immunoassays has made the quantification of multiple analytes obtained from clinical samples more feasible. However the variety of potential platforms and reagents has created challenges in selecting optimal assays for large-scale projects that demand consistent results and ultimately has hindered the use of these platforms in clinical research and their translation into clinical practice [25, 27, 40]. The lack of available data comparing multiplex platforms using consistent reagents makes it difficult to compare previous studies examining host biomarkers of severe infections. In this study, we evaluated two different multiplex platforms (Luminex® and Ella^TM^) using reagents from a single manufacturer (R&D Systems) to test over 400 well-annotated plasma samples from febrile children presenting with suspected severe infections. We found that the correlations between values for biomarkers of endothelial and immune activation tested within assay dynamic range were excellent. Moreover, using two different statistical methods, we identified consistent differences between absolute biomarker concentrations obtained using the two different multiplex platforms.

The selection of a multiplex immunoassay platform for a specific clinical project is based on many factors. When considering the optimal platform, we evaluated the availability of reagents for analytes of interest, the dilution factors to enable inclusion of multiple biomarkers in a single assay, the ease of assay performance, the time required to complete the assay with potential for translation to near-patient-care settings, as well as cost. Based on these criteria, the Luminex® [27] and Ella^TM^ [30] platforms were selected. Both platforms were reliable, with CV% for both platforms less than 5%, which is below the acceptable Food and Drug Administration (FDA) recommendations [41]. The Ella^TM^ platform has the advantage of being fully automated with internal triplicate sample testing, decreasing the sample and reagent quantity, and time required for assay performance. Although the per analyte cost was 5.8-fold greater for the Ella relative to the Luminex platform, the expense related to staff time for assay performance and data analysis could decrease this difference.

All of the biomarkers included in this study have been associated with severity of illness in severe infection [7–19]. Therefore, results of this study provide valuable data to guide future research in predictive and prognostic markers for life-threatening infections. In addition, several biomarkers evaluated here are noteworthy for their roles in infection-induced endothelial injury and as therapeutic targets for future clinical trials [42–44]. Validating a platform with minimal user interface and fast turn-around times would enable its use at near-patient-care to facilitate patient recruitment into novel clinical therapeutic trials.

Of note, there were significant differences observed in absolute biomarker concentrations between these two platforms. The bias towards higher concentrations with either the Luminex® or Ella^TM^ platforms, however, was uniform across the concentration ranges for most biomarkers as identified by the non-significant Pitman test p-values. This suggests that values of the biomarker concentrations were not different between the platforms at a different concentration range, making the relative values between the two tests comparable. Also worth noting is that although the absolute biomarker concentration values were statistically significantly different, the visual representation of the Log_e_-transformed biomarker concentrations displayed on Bland-Altman plots suggest that these concentrations were in a similar pg/mL range. The differences in absolute concentrations could be attributed to differences in the capture and/or detection monoclonal antibody specificity and/or affinity as described in **S3 Table**. Alternatively, competition between multiple monoclonal antibodies for antigen detection in the multiplex Luminex® assay could have been overcome with the Ella^TM^ platform multiplex design which uses independent microfluidic channels for each analyte. Finally, although both methods used reagents from R&D Systems and fluorescence was used as the indicator, the fluorochrome conjugated to streptavidin differed (Phycoerythrin (PE) for Luminex® and DyLight650 conjugate for Ella^TM^), affecting how the fluorochrome was excited, and what emission wavelength was detected by each platform, possibly contributing to a difference in concentration ranges on both lower and upper limits of quantification.

Selecting a platform to analyze large number of samples in a short time frame is best performed with an assay that can be done in multi-well format, enabling high throughput. Of the two platforms we tested, the Luminex® platform was better suited to this purpose. However, when near-patient or point-of-care data is required, with fast turn-around and with the capacity to test fewer samples, the Ella^TM^ platform offers the required characteristics. Our data provide evidence that concentrations obtained by both platforms are highly correlated. Therefore, it is possible to use either platform for clinical research examining a relationship between biomarker level and clinical end-point. However, given the statistically significant differences in absolute values, direct comparison of concentration values between platforms is problematic. These observations have implications for future point-of-care novel biomarker research as direct comparisons of data will likely only be possible if assays are performed using the same methodology with reagents obtained from the same manufacturer.

The strengths of this study include inclusion of biomarkers of current clinical interest in the field of critical illness and life-threatening infections. The large sample size of more than 400 patients provides this study with higher analytical power. Additionally, the selection of the Luminex® microsphere-based system, an established multiplex platform [27], as well as the novel Ella^TM^ microfluidics-based system [30] allowed the validation a novel platform with the potential to be used in future studies requiring real-time results. One of the clearly demonstrated strengths of the Ella^TM^ platform was a superior dynamic range compared to Luminex using reagents from R&D Systems.

This study had some limitations. First, frozen versus fresh plasma was used. Future studies are planned to validate biomarker concentrations quantified in fresh blood samples. Second, we could not analyze data obtained from the Ella^TM^ platform for sICAM-1 due to inappropriate sample dilution. This limitation highlights the limitation of multiplex assays which require all analytes tested to be at dilution suitable for the assay dynamic range. Also, up to 34% of concentration values quantified by the Luminex platform were below the limit of quantification (CHI3L1, sFlt-1, and IL-6). This limited the ability to compare the complete sample set, likely diminishing the accuracy of the comparison between the two platforms. This also limits the generalizability of the results to more critically ill patients, with higher biomarker levels.

Future clinical studies evaluating the potential of plasma markers as predictive and prognostic tools will require reliable, robust, easy to use multiplex platforms, providing data in real time at the point-of-care. Ultimately this approach could enable precision medicine via rapid triage of febrile patients and stratification for pathway-directed therapeutics for severe infections that may be applicable in critically ill patients identified in an emergency department or an intensive care unit.

In conclusion, plasma biomarkers of endothelial and immune activation can be reliably quantified using multiplex immunoassay-based platforms. The dynamic range of biomarker concentrations is greater for the Ella^TM^ platform. The intra-assay variability is excellent for both multiplex platforms. The trends in concentrations obtained by the Luminex and Ella instruments are highly correlated but the absolute value of analyte concentrations vary between these platforms. Novel multiplex assays that produce rapid and reliable results, with minimal user interface and enable near-patient application, could transform prediction of response to therapies, prognostication of clinical outcome, and risk-stratification for enrollment in clinical trials.

## Supporting information

S1 Table Dynamic range for biomarkers included in the Luminex® platform that did not overlap with Ella^TM^ multiplex platform. (DOCX)

S2 Table Inter-assay variability for a single healthy control quantified across 6 Luminex® assay plates and 6 Ella^TM^ cartridges. (DOCX)

S3 Table Comparison of reagents included in the Luminex® and Ella^TM^ platform. (DOCX)
