# Supplementary material for: Validation of two multiplex platforms to quantify circulating markers of inflammation and endothelial injury in severe infection
Source: PLoS One. 2017 Apr 18;12(4):e0175130. doi: 10.1371/journal.pone.0175130 (PMC5395141; doi:10.1371/journal.pone.0175130)
Supplement: S1 Table — (DOCX) [file pone.0175130.s001.docx]

**Validation of two multiplex platforms to quantify circulating markers of inflammation and endothelial injury in severe infection**

Aleksandra Leligdowicz^1,2^, Andrea Conroy^3^, Michael Hawkes^4^, Kathleen Zhong^1^, Gerald Lebovic^5^, Michael A. Matthay^6,7^, Kevin C. Kain^1,2*^

**Supporting Information**

**S1 Table.** Dynamic range for biomarkers included in the Luminex® platform that did not overlap with Ella^TM^ multiplex platform.

| **Biomarker** | **Dilution factor** | **Dynamic range (pg/ml)** |
| --- | --- | --- |
| **Cystatin C** | 30 | 21,363-5,295,787 |
| **Ang-1** | 3 | 8.2-97,077 |
| **IL-8** | 3 | 8.2-5,976 |
| **sTREM-1** | 3 | 131-95,530 |
| **Granzyme B** | 3 | 67-16,281 |
