# Supplementary material for: Validation of two multiplex platforms to quantify circulating markers of inflammation and endothelial injury in severe infection
Source: PLoS One. 2017 Apr 18;12(4):e0175130. doi: 10.1371/journal.pone.0175130 (PMC5395141; doi:10.1371/journal.pone.0175130)
Supplement: S2 Table — (DOCX) [file pone.0175130.s002.docx]

**Validation of two multiplex platforms to quantify circulating markers of inflammation and endothelial injury in severe infection**

Aleksandra Leligdowicz^1,2^, Andrea Conroy^3^, Michael Hawkes^4^, Kathleen Zhong^1^, Gerald Lebovic^5^, Michael A. Matthay^6,7^, Kevin C. Kain^1,2*^

**Supporting Information**

**S2 Table:** Inter-assay variability for a single healthy control quantified across 6 Luminex® assay plates and 6 Ella^TM^ cartridges.

| **Biomarker** | **Luminex (n=6)** | | **Ella (n=6)** | |
| --- | --- | --- | --- | --- |
|  | **Mean ±SD** | **CV%** | **Mean ±SD** | **CV%** |
| **sVCAM-1** | 619,741  ±69,713 | 10.7 | 766,369  ±150,640 | 19.7 |
| **sICAM-1** | 2,011,617  ±163,924 | 10.8 | 637,350  ±165,660 | 26.0 |
| **sTNFR-1** | 4,420  ±675 | 22.6 | 1,416  ±283 | 19.9 |
| **CHI3L1** | 11,000  ±0 | N/A | 19,712  ±3,415 | 17.3 |
| **Ang-2** | 2,086  ±675 | 22.3 | 1,542  ±276 | 17.9 |
| **sFlt-1** | 50  ±N/A | N/A | 92.6  ±16.0 | 17.3 |
| **IL-6** | 5  ±0 | N/A | 2.5  ±0 | N/A |
| **IP-10** | 70  ±19 | 30.0 | 108  ±7 | 6.6 |
