# Supplementary material for: Validation of two multiplex platforms to quantify circulating markers of inflammation and endothelial injury in severe infection
Source: PLoS One. 2017 Apr 18;12(4):e0175130. doi: 10.1371/journal.pone.0175130 (PMC5395141; doi:10.1371/journal.pone.0175130)
Supplement: S3 Table — (DOCX) [file pone.0175130.s003.docx]

**Validation of two multiplex platforms to quantify circulating markers of inflammation and endothelial injury in severe infection**

Aleksandra Leligdowicz^1,2^, Andrea Conroy^3^, Michael Hawkes^4^, Kathleen Zhong^1^, Gerald Lebovic^5^, Michael A. Matthay^6,7^, Kevin C. Kain^1,2*^

**Supporting Information**

**S3 Table:** Comparison of reagents included in the Luminex® and Ella^TM^ platform.

| **Biomarker** | **Recombinant Protein for Standard Curve** | **Capture monoclonal antibody** | **Detection monoclonal antibody** |
| --- | --- | --- | --- |
| **sVCAM-1** | Different | Different | Same |
| **sICAM-1** | Same | Same | Different |
| **sTNFR-1** | Same | Same | Same |
| **CHI3L1** | Same | Same | Same |
| **Ang-2** | Different | Different | Different |
| **sFlt-1** | Same | Different | Same |
| **IL-6** | Same | Same | Same |
| **IP-10** | Same | Different | Same |
